# Toward low-loss mid-infrared Ga_2_O_3_–BaO–GeO_2_ optical fibers

**DOI:** 10.1038/s41598-023-30522-1

**Published:** 2023-03-06

**Authors:** Théo Guérineau, Samar Aouji, Steeve Morency, Florian Calzavara, Patrick Larochelle, Philippe Labranche, Jerome Lapointe, Sylvain Danto, Thierry Cardinal, Evelyne Fargin, Martin Bernier, Réal Vallée, Younès Messaddeq

**Affiliations:** 1 Center for Optics, Photonics and Lasers (COPL), Université Laval, Québec, G1V 0A6 Canada; 2 ICMCB, UMR 5026, Université de Bordeaux, CNRS, Bordeaux INP, 33600 Pessac, France

**Keywords:** Materials science, Materials for optics, Fibre lasers

## Abstract

The development of efficient and compact photonic systems in support of mid-infrared integrated optics is currently facing several challenges. To date, most mid-infrared glass-based devices are employing fluoride or chalcogenide glasses (FCGs). Although the commercialization of FCGs-based optical devices has rapidly grown during the last decade, their development is rather cumbersome due to either poor crystallization and hygroscopicity resilience or poor mechanical-thermal properties of the FCGs. To overcome these issues, the parallel development of heavy-metal oxide optical fiber from the barium-germanium-gallium oxide vitreous system (BGG) has revealed a promising alternative. However, over 30 years of fiber fabrication optimization, the final missing step of drawing BGG fibers with acceptable losses for meters-long active and passive optical devices had not yet been reached. In this article, we first identify the three most important factors that prevent the fabrication of low-loss BGG fibers i.e., surface quality, volumic striae and glass thermal-darkening. Each of the three factors is then addressed in setting up a protocol enabling the fabrication of low-loss optical fibers from gallium-rich BGG glass compositions. Accordingly, to the best of our knowledge, we report the lowest losses ever measured in a BGG glass fiber i.e., down to 200 dB km^−1^ at 1350 nm.

## Introduction

Following the outstanding development of low-loss silica fibers in the 1970s, the emergence of high-speed long-haul telecommunication systems and high-power fiber lasers have revolutionized our daily lives^1,2^. However, silica fibers do not transmit light above 2.5 μm and thus cannot be employed for applications in the so-called mid-infrared (MIR) domain^3^. As a result, complementary MIR-transmitting glass families have been discovered and developed, including tellurite, chalcogenide, fluoride and germanate glasses. The development of fluoride fibers has somehow overcome most of the other MIR glass families, with a wide range of fibers now commercially available. Although the fluoride glasses expand over a large choice of glass compositions, including the zirconium fluoride, indium fluoride or aluminum fluoride families, these soft glasses possess a low glass transition temperature (Tg), while their reduced thermal/mechanical/chemical stability compared to other MIR glasses makes their handling more challenging^3,4^.

Among other MIR glasses, germanate glasses are one of the best alternatives to fluoride glasses in terms of thermal and mechanical properties. Indeed, their Tg can reach 700 °C, their optical transmission windows can span from 0.28 up to 5.5 μm and their Knoop micro-hardness can extend up to 5.1 GPa^5^. To date, minimal germanate losses (200 dB km^−1^) were obtained in lead-germanate glasses^6^. However, the presence of lead oxide in the glass composition contributes to the downgrade of both thermal and mechanical properties, i.e., Tg below 400 °C and Vickers hardness down to 2.5 GPa^7^, while restricting their use in various application fields due to severe worldwide regulations on lead-containing products.

Since the discovery of the barium-gallium-germanium (BGG) glasses in the 1990s^8^, considerable efforts have been deployed to further enhance the glass properties^9–13^, draw them into fibers^14–16^ and also functionalize them^17–19^. In the meantime, gallium-rich, namely gallate, BGG glasses (GaO_3/2_/GeO_2_ ratio in mol% larger than 1) have attracted significant attention, since their thermal, optical and mechanical properties are even superior to those of germanate-based BGG compositions (GaO_3/2_/GeO_2_ ratio in mol% smaller than 1). Indeed, the substitution of Ge^4+^ for Ga^3+^ ions increases both optical transmission window up to 6.0 μm and Knoop micro-hardness up to 5.4 GPa, while the solubility of rare-earth ions remains high (more than 10 mol%)^5,20,21^.

Hence, BGG glasses do not excel with one specific property, as fused silicate with their high Tg and crystallization resilience or chalcogenide with their optical transmission up to the far-infrared domain. However, it is the combination of their glass transition temperature, hardness, optical transmission, mechanical resistance and rare-earth solubility, that makes BGG glass fiber extremely valuable in the future development of both passive and active optical mid-IR fibers.

In the groundbreaking endeavor for the development of robust and reliable BGG fibers, two of the three major milestones have been achieved: the manufacture of a core-clad fiber and the reduction of the OH group content^12,22,23^. However, the last but not least milestone, consisting in reducing the background optical losses below the dB per meter, has not been achieved yet, while the predicted optical losses should reach a few dB per kilometer^24^.

In this article, we report the fabrication of low-loss gallium-rich BGG glass fibers produced by the preform-to-fiber approach. By identifying and solving the three most predominant factors that prevent the manufacture of low-loss BGG fibers, i.e., surface quality, volumic striae and fiber thermal-darkening, we successfully drew into tens-of-meters-long segments the very first BGG fiber with optical losses down to 200 dB km^−1^ at 1350 nm. Furthermore, we also drew into fiber a low-OH BGG preform presenting fair background losses up to the fundamental absorption of water, i.e., 2800 nm. Hence, our work brings the last missing milestone to the development of BGG fibers of practical use.

## Methods

### Material fabrication and fiber drawing of the selected gallate glass composition

All glass precursors (at least 99.99% of purity) are precisely weighed, mixed and introduced into a platinum crucible.

For Preforms A and D, the mixture is melted at 1500 °C in air for 1.5 h, while every 30 min the crucible is removed from the furnace and stirred. For Preform B, the mixture is melted at 1500 °C in air for 1.5 h then cooled down at 1450 °C just before casting. For Preform C, the mixture is melted at 1600 °C in air for 1.5 h, while every 30 min the crucible is removed from the furnace and stirred.

For all preforms, a stainless-steel mold is preheated 50 °C below the glass transition temperature. When the mold is thermalized, the glass melt is quickly poured inside the mold. Then, the glass preform is annealed at 50 °C below the Tg for 5 h and slowly cooled down to room temperature. The annealed Preforms B, C and D undergo a polishing process on a homemade apparatus designed for cylindrical preform, using successive grit size steps down to a 1 μm cerium oxide slurry.

For the glass preform containing a very low OH content, the melting and casting processes are performed under an argon atmosphere. Also, during the glass melting, 3% in weight of ammonium bifluoride is added to the glass precursors as a dehydrating agent. The remaining fluorines in the glass decrease by 60 °C the glass transition temperature and by 2 × 10^–3^ the refractive index, compared to the values presented in Table 1.

**Table 1:** Physico-chemical properties of the selected gallate composition performed on its preform form: theoretical and experimental compositions measured by induced coupled plasma—optical emission spectroscopy, glass transition and crystallization temperatures and refractive index measured at 1538 nm.

GaO_3/2_ atomic %		GeO_2_ atomic %		BaO atomic %		YO_3/2_ atomic%		Tg (± 3 °C)	Tx (± 3 °C)	n at 1538 nm
Theo	Exp. (± 1%)	Theo	Exp. (± 1%)	Theo	Exp. (± 1%)	Theo	Exp. (± 1%)	718	905	1.74
42.0	39.7	27.0	27.9	30.0	31.3	1.0	1.1			

Preforms are inserted inside a furnace under a dinitrogen atmosphere. At around 820 °C, the fiber drawing is initiated. As no polymer is coated on the glass fiber, the fiber is carefully wound manually on a capstan.

### Bulk and fiber characterization

Differential scanning calorimetry (DSC) measurements were performed with a DSC 404 F3 Pegasus calorimeter at a heating rate of 10 °C min^−1^. Thanks to DSC measurements, the glass transition temperature was extracted. Chemical analyses were conducted by electron-probed microanalysis (EPMA) on a CAMECA-SX100 apparatus. Wavelength Dispersive Spectroscopy (WDS) was acquired to measure the cationic elements, with an average value based on 8 acquisitions. The refractive indices were measured at five different wavelengths (532 nm, 632.8 nm, 972.4 nm, 1308.2 nm and 1537.7 nm) with a prism coupler refractometer (Metricon, 2010/M). The UV–visible–near-IR transmission spectra from 200 to 1100 nm were recorded on a Cary 60 UV–Vis (Agilent) spectrometer by steps of 1 nm, while the near-IR-MIR transmission spectra were obtained from 1 to 7 μm using a Fourier-transform infrared spectrometer with an average of 50 scans and a resolution of 4 cm^−1^. Raman spectra were recorded at room temperature from 200 to 1100 cm^−1^ using a Renishaw inVia Raman microscope and a 50X microscope objective. A continuous wave laser operating at 633 nm was used for excitation. Scanning electron microscopy in back-scattered electron mode was carried out on a Quanta 3D (FEI) (15 kV and low vacuum) equipped with a 10 mm^2^ Si(Li) crystal detector (Ametek) for the EDX measurements. X-ray scatterings were recorded at room temperature and collected on a Panalytical AERIS diffractometer equipped with an X’celerator detector over an angular range of 2θ = 10–80°. The Cu-Kα radiation was generated at 40 kV and 8 mA (lambda = 0.15418 nm).

The propagation losses of the fibers were measured by the cut-back method. Measurements were performed using a fiber supercontinuum source (superK compact de NKT photonics, a monochromator (Bruker) and a PDA10CS from Thorlabs detector sensitive in the infrared range (700–1800 nm) for the glass fiber with low losses. For MIR fiber loss characterization of the low OH content, a home-made fluoride supercontinuum source operating from 1000 to 3900 nm was used together with a Yokogawa AQ6376 OSA covering from 1500 to 3400 nm. All fiber cleaves were made using a Vytran LDC401A cleaving system and methodically inspected with a microscope objective.

## Result and discussion

### Glass composition choice

The preform-to-fiber drawing method requires a glass composition with strong resilience to the devitrification process. In fact, the formation of crystals either at the surface or in the bulk of the drawn fiber is resulting in poor optical and mechanical properties^25^. As a rule of thumb, the temperature difference (ΔT) between the crystallization (Tx) and the glass transition (Tg) temperatures is a convenient glass stability indicator that is generally expected to be larger than 100 °C. However, this indicator is somehow misleading with respect to the glass surface devitrification of the BGG compositions^15,16,25^. Avoiding alkaline ions as well as adding lanthanide oxides, i.e., Y_2_O_3_, La_2_O_3_, Yb_2_O_3_, Gd_2_O_3_, etc., in BGG glass compositions has proven to be a very effective mean to enhance the resilience to surface devitrification^13,15,16,26,27^. Nonetheless, the addition of lanthanide elements also significantly increases both glass melt viscosity and casting temperature. Hence, from a vast assortment of BGG compositions that we have synthesized, we have selected the following gallate composition enabling a minimal addition of yttrium oxide while providing superior glass stability (Table 1).

To vitrify gallate glasses, the formation of trivalent gallium ions in a tetrahedral structural unit must be favored against higher coordination sites. However, gallium tetrahedral units possess a negative charge [GaO_4_]^−^ which requires to be compensated. In the selected glass composition, the quantity of Ba^2+^-providing positive compensation charges is almost one and a half greater than the number of gallium ions.

As depicted in Table 1, the elemental composition difference is neglectable and seems to even indicate a smaller Ga/Ba ratio (1.27) than expected (1.4). Hence, the formation of [GaO_4_]^−^ in the selected gallate composition is strongly favored against five- and six-fold coordinated sites.

Performed by differential scanning calorimetry on ground powder, the characteristic temperatures Tg and Tx were measured as 718 and 905 °C, respectively. Additionally, while the glass presents a high Tg and a large ΔT (≈ 190 °C), the ideal casting temperature occurs at a lower temperature than for germanate-rich BGG compositions.

**Figure 1:**
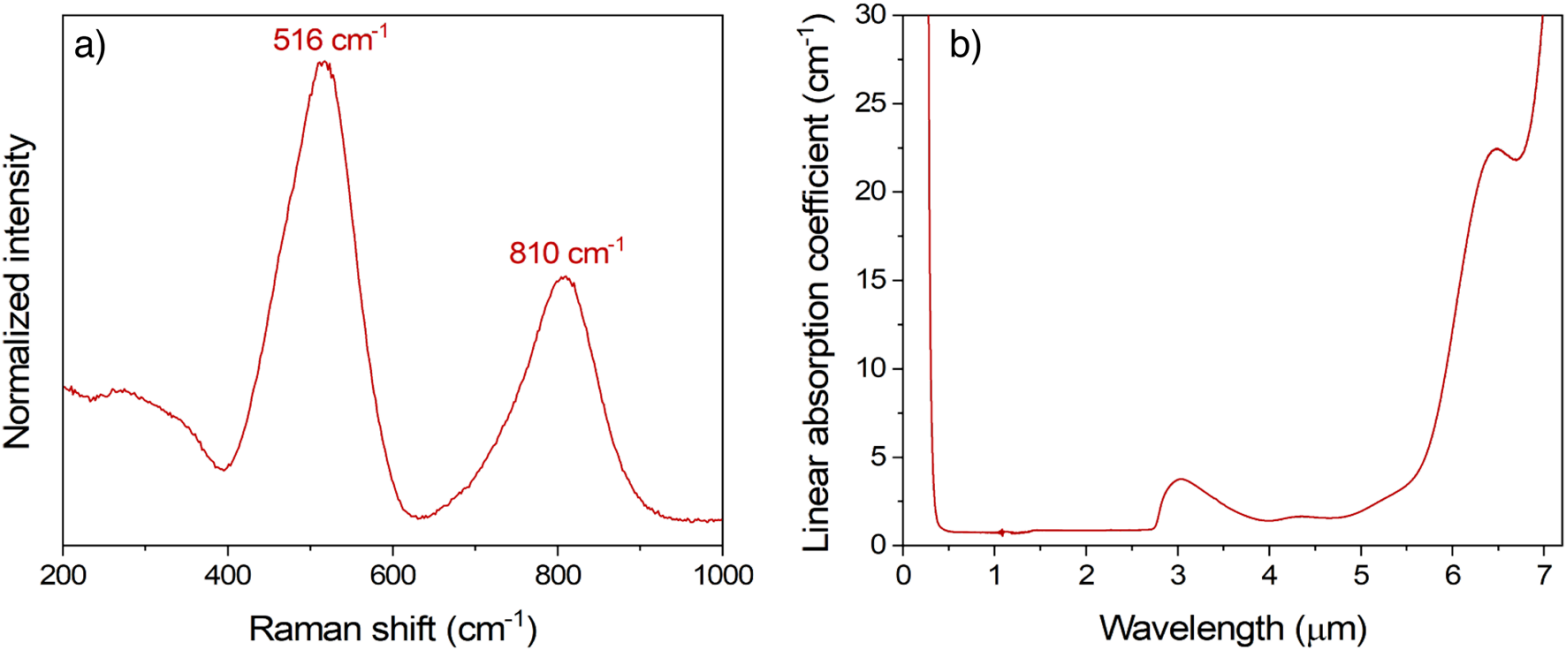
(**a**) Raw data Raman spectrum normalized at their maximum intensity measured at 633 nm; (**b**) Linear absorption coefficient in the UV–visible-to-Mid-IR wavelength range for the selected gallate composition.

In Fig. 1 are represented both the Raman spectrum and linear absorption coefficient evolution for the selected gallate composition. The Raman spectrum can be separated into three regions: high (650–1000 cm^−1^), intermediate (400–650 cm^−1^) and low (200–400 cm^−1^) frequencies. The highest spectral domain can be attributed to symmetric and antisymmetric stretching modes of gallium and germanium tetrahedral units [TO_4_]^16,28^. The intermediate spectral range can be assigned to several vibrational contributions of T–O–T bending with in-the-plane T–O–T oxygen motions^28,29^. Finally, the lowest spectral domain can be assigned to either out-of-plane Oxygen motions in bent T–O–T bridge (T = Ge or Ga in four-fold coordination)^28^ or network-modifying cations vibrating in large interstitial sites^29^.

In the selected composition, the predominant Raman response is pointing at 516 cm^−1^ with a shoulder at around 450 cm^−1^ (barely showing in Fig. 1). As the amount of gallium is one and a half that of germanium, the changeover of gallium and germanium tetrahedral units in the glass skeleton should follow the same pattern. Thus, the formation of two linked [GeO_4_] entities is likely occasional which explains the minimal band contribution at 450 cm^−1^, usually assigned to the specific Ge–O–Ge bridges, while having the predominant response at 516 cm^−1^ is very consistent with a glass structure rich in [GaO_4_]^−^^29^. As reported in Table 1, the quantity of positive-charge compensators brought by the Ba^2+^ ions is more than necessary to stabilize the [GaO_4_]^−^ units. Hence, the remaining Ba^2+^ ions which do not contribute to the [GaO_4_]^−^ compensation mechanism (approximately a third of the total barium content) are depolymerizing the glass network leading to the apparition of Ge∅_3_O^−^ entities. Ge∅_3_O^−^ entities are GeO_4_ tetrahedral units with three bridging oxygens and one non-bridging oxygen symbolized by ∅ and O^−^ symbols, respectively. The second most intense contribution is peaking at 810 cm^−1^ which can be explained by the presence of non-bridging oxygens on the germanium tetrahedral units Ge∅_3_O^−^^20^.

The linear absorption coefficient evolution has been measured from the UV to the mid-infrared. As observed in Fig. 1b, the optical window transparency of the selected composition, as defined at 10 cm^−1^, spans from 300 nm, i.e., bandgap tail absorption, up to 5.9 μm, i.e., multiphonon absorption. Compared to germanate-rich BGG glasses, the mid-IR edge is redshifted by about 200 nm thanks to the low germanium content in our gallate composition. The presence of hydroxyl absorption bands is reported between 2.7 and 5 μm as no special care was taken to remove it during the glass synthesis^30^.

### Identification of the main detrimental factors

We have identified three main detrimental factors preventing the drawing of low-loss BGG fibers: surface quality, volumic striae and fiber darkening.

To isolate each factor, four preforms were fabricated, namely A, B, C and D, respectively (refer to Experimental section for more details): an unpolished preform with great bulk homogeneity (to illustrate surface quality issues), a polished preform with poor bulk homogeneity (to illustrate volumic striae issues), and two polished preforms with great bulk homogeneity but cast either at 1600 °C (to illustrate fiber thermal-darkening issues) or at 1500 °C (producing the low-loss BGG fiber). For clarification, the terms “polished” or “unpolished” refer here to the entire polishing of the preform surface. For more details on the preform preparation protocol, readers are invited to refer to the Experimental section.

### Surface quality

**Figure 2:**
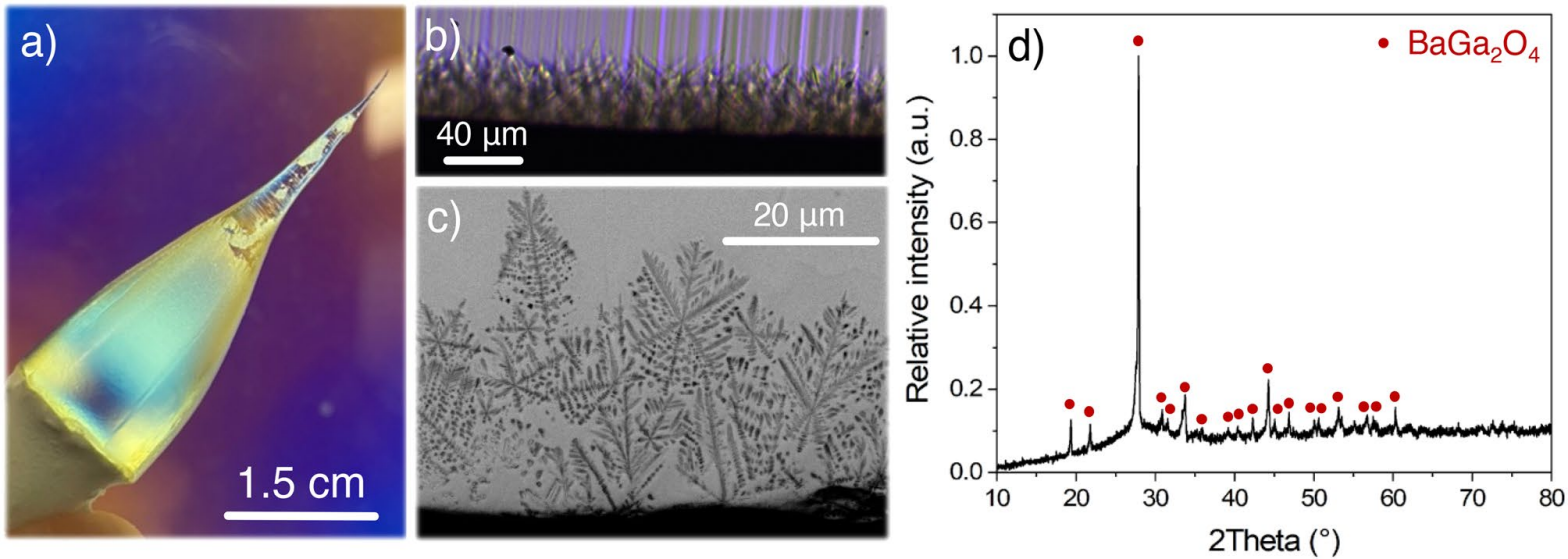
(**a**) Neckdown of unpolished preform observed through a polariscope; Edge of the preform neckdown transection imaged by (**b**) an optical microscope in transmission mode and (**c**) a scanning electron microscope in backscattered electron mode; (**d**) Diffractogram of the preform neckdown surface.

As previously introduced, one of the main issues faced during the fiber drawing of BGG compositions through the preform-to-fiber method is the glass devitrification occurring at both the preform and fiber surfaces. Such surface crystallization prevents the fiber to be mechanically robust, while in single-index fibers it also significantly increases the optical losses at the core-cladding interface, i.e., glass-air or glass-coating, rich in defects. As explained by Zanotto, “the surface nucleation is mainly due to impurity particles whose numbers are inversely proportional to the degree of surface perfection and cleanliness”^31^. Hence, to illustrate this issue, the neckdown characterizations of the unpolished preform are shown in Fig. 2.

First, we note that the neckdown geometry is irregular. In parallel to this, the crystallization process is resulting in the formation of a 40 μm-thick layer. Using both optical and electronic imaging, the crystallization morphology has been analyzed (Fig. 2b,c). It revealed a crystal organization between ordered dendrites with crystallographic symmetry and disordered polycrystalline dendrites, indicating a growth rate mostly governed by heat and material diffusion^32^. It suggests that the growth rate is relatively faster than the ion diffusion. In the meantime, the diffractogram of the crystallized layer has been monitored and revealed the formation of a single phase attributed to hexagonal P6_3_ BaGa_2_O_4_ polycrystals. Such crystal composition is coherent with the formation of dendrites since this composition differs strongly from the glass stoichiometry and requires the diffusion of the low-mobility germanium ions.

Consequently, since the preform was not polished prior to the fiber drawing, the poor preform surface quality promoted surface nucleation. As long as the surface nucleation is prevented notably by the careful preparation of the glass preform through both polishing and cleaning processes, no crystallization was detected during the fiber drawing process.

### Volume quality

**Figure 3:**
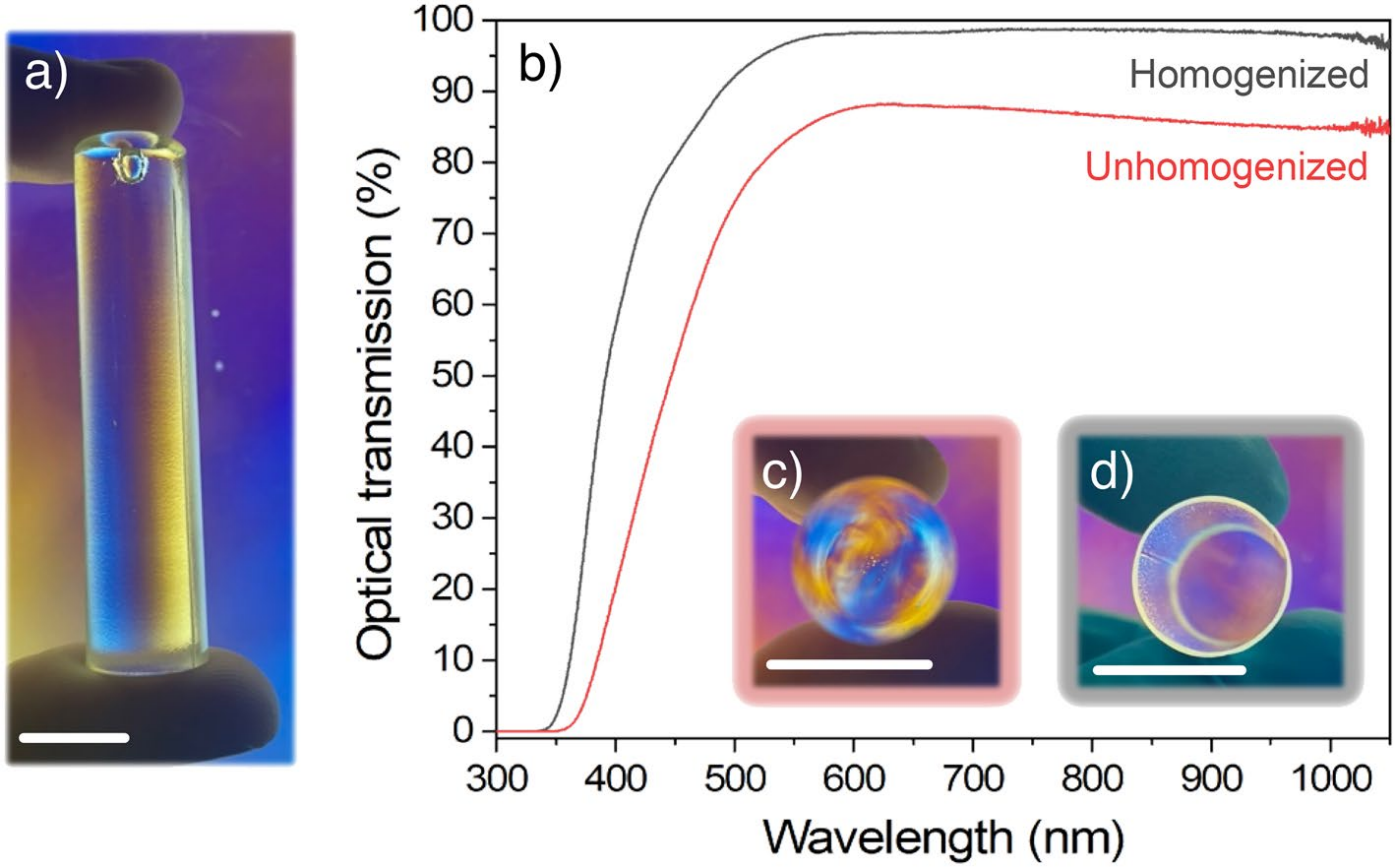
(**a**) Photograph of an as-casted glass preform of the selected gallate composition observed through a polariscope; (**b**) UV–visible optical transmission with Fresnel loss correction measured along each glass preform with two polished opposite faces; (**c**) Photograph of these latter two preforms with either a (**c**) poor or (**d**) great bulk homogeneity observed through a polariscope. Scale bars: 1.5 cm.

The propagation of optical modes along the glass fiber is very sensitive to the presence of striae inside the glass preform, which could originate either from a poor glass melt homogenization and/or an inappropriate melt casting method^33^. Several characterization techniques were developed over the years to assess the glass’ optical quality after melt casting based on various evaluations: shadowgraph, striaescope, interferometry or polariscope^14,34^. In this study, we have chosen the polariscope for its practicality and effectiveness. Two preforms either unhomogenized or homogenized via optimized casting temperature and stirring are compared in Fig. 3.

The visual inspection of the typical as-casted preform of the selected gallate composition, as shown in Fig. 3a, does not allow the detection of severe bulk inhomogeneities or bubbles. However, to confirm the melt-quenching method’s appropriateness a careful optical examination must be carried out along the axis of the preform. Hence, both preform ends are optically polished and then inspected through a polariscope as presented in Fig. 3c,d. On the first hand, in the preform with unoptimized melt-quenching parameters (Fig. 3c), wide striae are unambiguously frozen in the volume, which leads to a significant attenuation of the optical transmission, even after Fresnel loss correction (determined for a refractive index of 1.745 at 972 nm) (Fig. 3b-red curve). On the other hand, the preform with optimized melt-quenching parameters reveals a clear glass with very few striae (Fig. 3d) which explains the great optical transmission (Fig. 3b-black curve) above 98% from 580 nm up to 1100 nm.

Unsurprisingly, the drawing of the preform with poor bulk homogeneity results in a lossy optical fiber. Indeed, even though the preform is heated above the glass transition temperature during the fiber drawing process, the temperature and time of exposure are not high and long enough, respectively, to enable striae relaxation.

**Figure 4:**
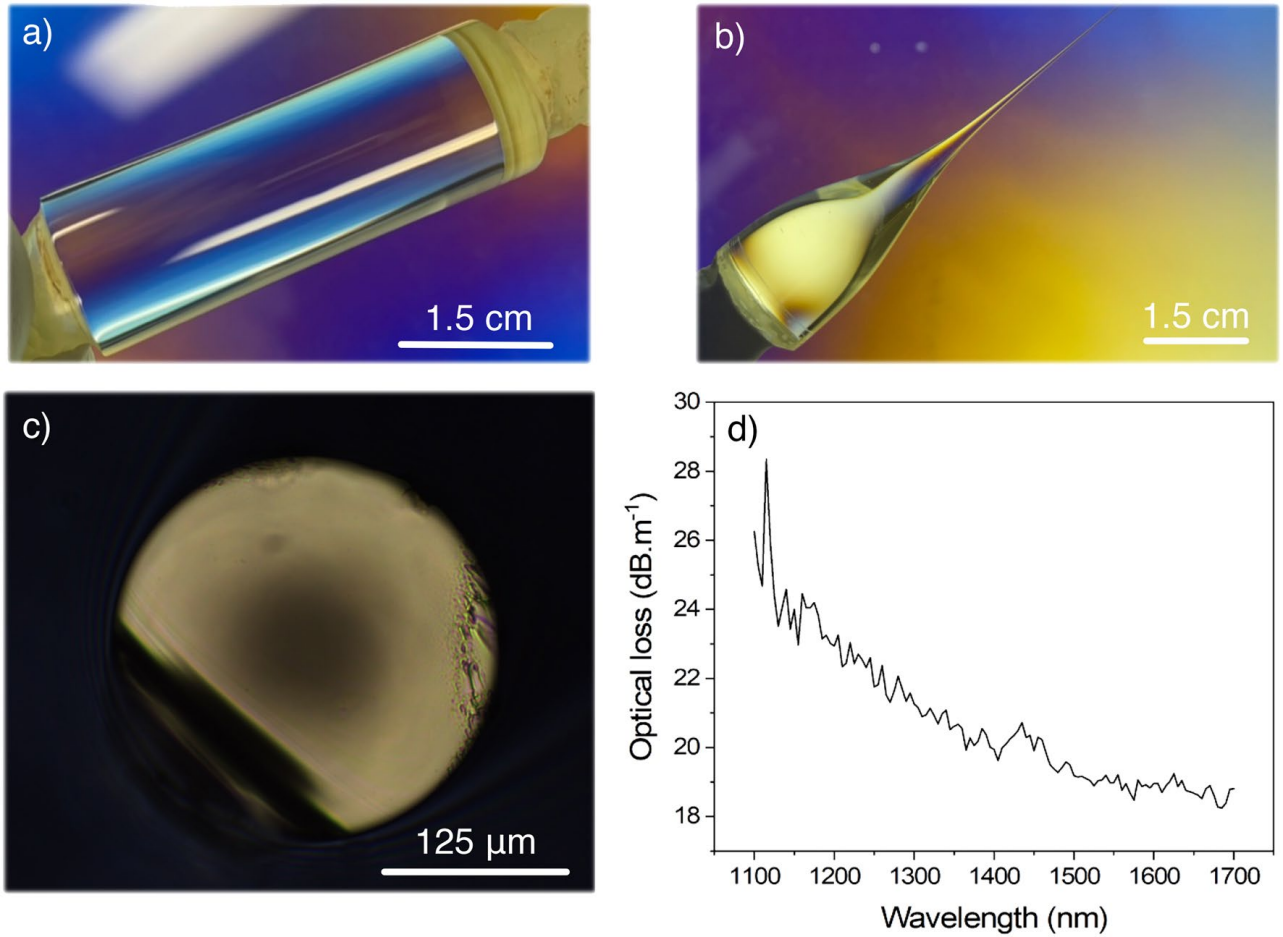
Photograph of the as-polished preform cast at 1600 °C observed through a polariscope (**a**) before and (**b**) after fiber drawing; (**c**) Optical imaging in transmission mode of the fiber transection presenting the darkening phenomenon; (**d**) Optical losses measured in a 5 m-long darkened fiber.

Performing the melt casting at higher temperatures is expected to reduce both detrimental stria occurrence and size. Consequently, increasing melt casting temperature should be beneficial from this viewpoint. The optical images before and after fiber drawing of the polished preform with great bulk homogeneity and cast at 1600 °C are shown in Fig. 4 along with loss measurement of the drawn fiber.

As observed in Fig. 4a, the as-polished preform does not present any visible detrimental surface crystallization or volume striae. Upon drawing into tens-of-meters-long fiber segments, the preform neckdown also remains defect-free while its geometry (Fig. 4b) suggests well-adjusted drawing parameters. Whereas the fiber appears to be mechanically robust with respect to twist, curvature and tension, its optical properties were far from optimum. Indeed, the optical analysis carried out on an optical microscope has revealed a darkening phenomenon in the fiber’s inner region. The observation of such a darkening area was absent in the glass preform. The presence of a germanium-related defect, i.e., thermally unstable defect, could have been a possibility but a heat treatment at 400 or 600 °C does not reduce the darkening, while no orange luminescence under UV excitation is observed either^35^. Sontakke and Annapurna have investigated the formation of gallium or germanium nanoparticles in a close glass family composed of Ga_2_O_3_, GeO_2_, BaO, CaO, MgO and La_2_O_3_. Their study has revealed a strong correlation between the appearance of an absorption band in the entire visible region with a maximum at 450 nm and the formation of gallium or germanium nanoparticles^36^. In the meantime, an increase in the melt casting temperature is often associated with a more oxidizing environment, which for heavy-metal oxide glasses tends to considerably increase the level of platinum attack^37^. In our BGG fiber, the presence of gallium, germanium or platinum nanoparticles could not be confirmed by transmission electron microscopy yet. However, the optical losses were measured via the cut-back method on a 5 m-long fiber from 1100 to 1700 nm. As depicted in Fig. 4d, the attenuation spectrum reveals a significant background loss in excess of 20 dB m^−1^, decreasing over the whole spectral region. Such trend would be coherent with the appearance of a surface plasmon resonance due to metallic nanoparticles, but also to their associated Rayleigh scattering.

### Appropriate glass fabrication

**Figure 5:**
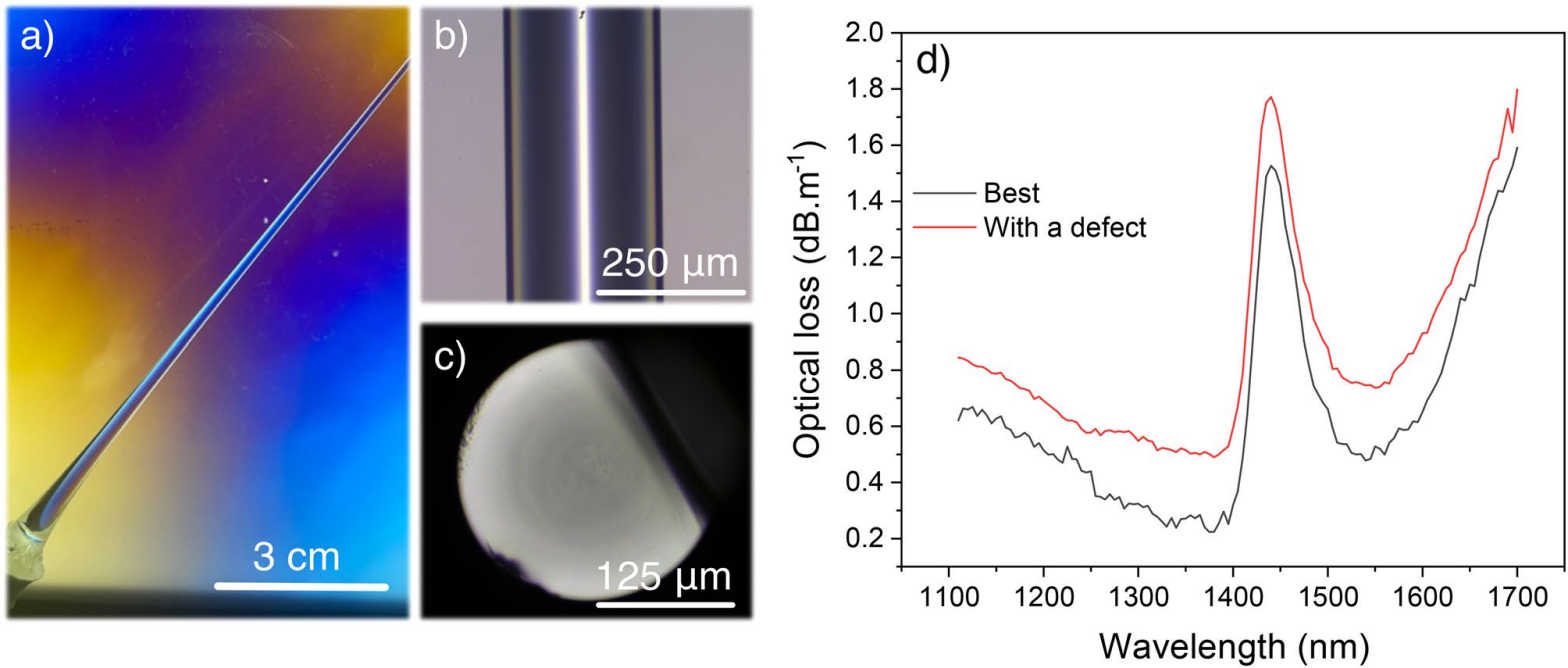
(**a**) Photograph of the as-drawn preform drop with appropriate fabrication parameters observed through a polariscope; Optical imaging in transmission mode of (**b**) the fiber and (**c**) its transection; (**d**) Optical losses measured in a 10 m-long fiber.

The optical images and loss measurements upon fiber drawing of the polished preform with optimized melt-quenching parameters and cast at 1500 °C are presented in Fig. 5.

The as-drawn preform drop is shown in Fig. 5a and does not present any visible defect, such as crystallization spots or layers. From the single glass preform, 40 m of 250 μm-diameter fiber were drawn with very few surface defects. The longest drawn fiber segment with non-detrimental surface defects is 20 m long. Most of the drawn fibers are defect-free as presented in Fig. 5b,c. Several attenuation measurements were carried out to assess the entire fiber quality. Unsurprisingly, fiber segments with surface defects presented higher losses up to a few dB m^−1^. In Fig. 5d are represented the optical losses measured in two 10-m-long fiber segments from 1100 to 1700 nm with a 3-m cut-back for each. Our best fiber segment (Fig. 5d—black curve) displays losses down to 200 dB km^−1^ at about 1350 nm. Additionally, a significant absorption band at 1450 nm and a continuous increase of the optical losses beyond 1400 nm are observed.

Molecular water possesses several absorption bands in the near-infrared optical domain, notably with an intense band at 1960 nm and a moderate one at 1445 nm^38,39^. From Tsubomura’s work, the 1960 nm band observed in liquid water was assigned to a combination of the fundamental OH stretch tone with a deformation mode. In the meantime, as reported by Wang et al. in lead-germanate glass fibers, an overtone band of the 2.9 μm fundamental absorption of hydroxyl groups is located at 1450 nm, while they observe similarly a continuous increase of the optical losses beyond 1400 nm when no dehydration process is carried out on their glass preform^40^. Throughout their dehydration study, Wang et al. have highlighted that hydroxyl purification decreases the 1450 nm band while flattening the background optical losses beyond 1400 nm. Finally, similar results were also reported for tellurite glasses^41,42^.

As no special care was taken to dehydrate our glass preform, both the 1450 nm band and the continuous absorption beyond 1400 nm are attributed to the presence of OH groups inside the glass fiber. Hence, even though the presence of OH groups hides the real background losses of BGG fibers, the authors truly expect that the background losses are even lower than 200 dB km^−1^ at 1350 nm and at higher wavelengths considering the Rayleigh scattering. Indeed, as reported on the BGG transmission spectrum (Fig. 1b), no additional absorptions are present until the first multiphonon contribution above 5 μm.

Additionally, considering optical attenuations measured on 10-m-long single-index fibers, if the glass composition was chemically sensitive to water or hygroscopic, the optical attenuations would have been much higher than those measured, confirming the greater BGG resilience to water than fluorides.

The optical attenuation measurement was also performed on a fiber segment with a small surface defect (Fig. 5d—red curve). As expected, the background losses are higher than without surface defects and are down to 500 dB km^−1^. Nonetheless, both general spectral distribution and intensity of the OH group overtone band are very similar to that one without surface defect, confirming the good quality of the loss measurement as well.

In that respect, we demonstrate the fiber drawing of tens-of-meter-long BGG fibers with both excellent optical and surface qualities. Indeed, even though the presence of OH and/or water increases artificially the background losses, it is the first time that optical attenuations in BGG fibers are measured below the dB per meter, down to 200 dB km^−1^. Those propagation losses in a BGG fiber augur well for the manufacture of meter-long active and passive optical devices, which are in a growing demand for Mid-IR applications^43–45^, when the same background losses will be obtained in the entire domain of the BGG transparency.

**Figure 6:**
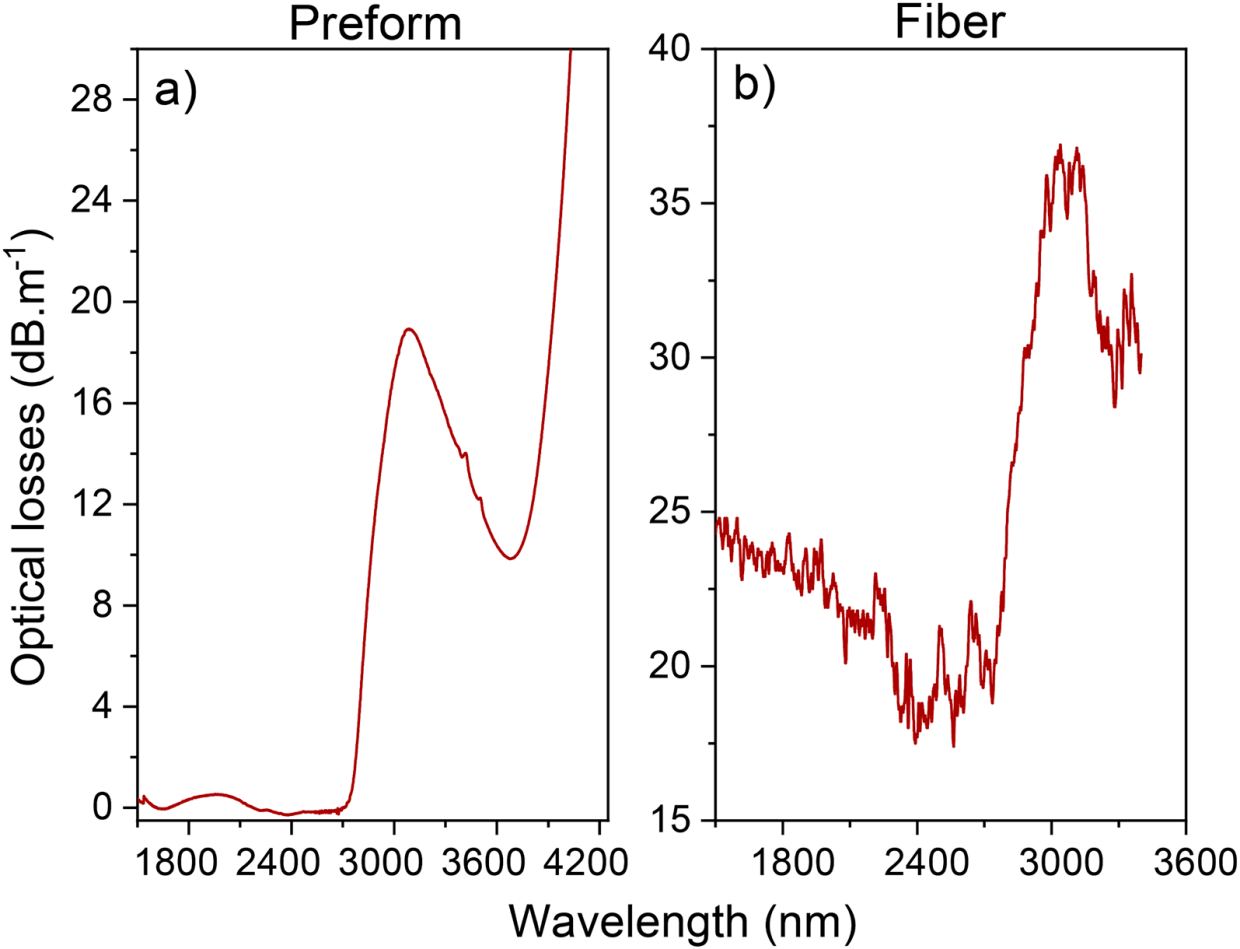
Optical losses measured for a low-OH-containing (**a**) preform determined from a FTIR measurement and (**b**) fiber drawn from the corresponding dehydrated preform.

### Dehydrated glass preform and fiber

In Fig. 6 are presented the optical loss measurements for a dehydrated BGG preform and its as-drawn fiber. Because of the dehydration process, the concentration of hydroxyl group content has been considerably diminished up to optical losses of 19 dB m^−1^ at 2900 nm, while they were two orders of magnitude higher in the hydrated one. In the counterpart drawn fiber, by subtracting the background losses of around 17–20 dB m^−1^ in the range 2400–2600 nm, the measured optical OH losses are around 17 dB m^−1^, which confirmed the optical fiber losses due to the OH content can be predicted from an FTIR measurement performed into the preform. This also confirms that the employed dehydration process is highly efficient and that there is no significant OH contamination in the glass during the fiber drawing process as expected. Due to the difficulties to well homogenize the glass melt during the melting process, high background lossesQ4 are recorded. Nonetheless, for the first time in BGG fibers, the spectral distribution without other contributions than fundamental absorption of OH groups has been successfully measured, with material losses up to 3400 nm.

## Conclusion

In conclusion, the three actual detrimental factors countering the fiber drawing of low-loss barium-gallium-germanium glasses have been clearly identified: surface quality, volumic striae and thermal darkening. While both surface quality and volumic striae are basic factors well-known in the glass community, the darkening of BGG glass fibers occurring throughout the fiber drawing process has been evidenced. For each detrimental factor highlighted in the BGG glass system, a solution has been elaborated to reach, for the first time, acceptable losses (down to 200 dB km^−1^) compatible with the manufacture of meter-long active and passive Mid-IR optical devices. The obtained results offer a clear protocol to fabricate low-loss optical fiber from BGG glass composition. When achieved in core-clad hydroxyl-free BGG glasses, these low losses will open up new avenues for the development of new fiber-based components and lasers operating above 2.2 μm.

## Data Availability

The datasets used and/or analysed during the current study are available from the corresponding author on reasonable request.
